# Transcranial Direct Current Stimulation (tDCS): A Beginner's Guide for Design and Implementation

**DOI:** 10.3389/fnins.2017.00641

**Published:** 2017-11-22

**Authors:** Hayley Thair, Amy L. Holloway, Roger Newport, Alastair D. Smith

**Affiliations:** ^1^School of Psychology, University of Nottingham, Nottingham, United Kingdom; ^2^School of Psychology, University of Plymouth, Plymouth, United Kingdom

**Keywords:** transcranial direct current stimulation, brain stimulation, protocol, cortical modulation, anodal, cathodal

## Abstract

Transcranial direct current stimulation (tDCS) is a popular brain stimulation method that is used to modulate cortical excitability, producing facilitatory or inhibitory effects upon a variety of behaviors. There is, however, a current lack of consensus between studies, with many results suggesting that polarity-specific effects are difficult to obtain. This article explores some of these differences and highlights the experimental parameters that may underlie their occurrence. We provide a general, practical snapshot of tDCS methodology, including what it is used for, how to use it, and considerations for designing an effective and safe experiment. Our aim is to equip researchers who are new to tDCS with the essential knowledge so that they can make informed and well-rounded decisions when designing and running successful experiments. By summarizing the varied approaches, stimulation parameters, and outcomes, this article should help inform future tDCS research in a variety of fields.

The enhancement of human cognitive processes has long been a focus of scientific experimentation, and transcranial direct current stimulation (tDCS) has recently come to the fore as a promising tool for modulating cognitive and motor skills (Nitsche and Paulus, 2000). Popularity of the technique has grown over the past decade, as exemplified in a PubMed search, returning 1,500 published articles containing the phrase “tDCS” between 2011 and 2015, in comparison to just 65 articles published between 2000 and 2005. tDCS involves the emission of a weak electrical current, traditionally via the placement of two electrodes attached to the scalp of a participant. In this traditional, unihemispheric tDCS set-up, one electrode is known as the target electrode, and the other the reference electrode. Some montages place the reference electrode extracephalically, for example on the upper arm. On the other hand, electrodes may be placed “bihemispherically” to emit dual stimulation to two parallel cortices (e.g., the parietal cortices—Benwell et al., 2015). This refers to purposefully upregulating one region of the brain, while downregulating another (Lindenberg et al., 2010). It is also now becoming common to use several smaller electrodes, rather than a singular target and reference electrode (see section What Size should the Electrodes Be?).

During stimulation, current flows between the electrodes, passing through the brain to complete the circuit. It is generally assumed that a positive anodal current temporarily facilitates behaviors associated with the cortical region under the target electrode, whereas a negative cathodal current inhibits behaviors (Nitsche et al., 2008). Like transcranial magnetic stimulation (TMS), active stimulation can be compared with a sham protocol (see section What Is a Sham Condition?). Direction of current flow differentiates anodal and cathodal stimulation by modulating the resting membrane potential of the neurons stimulated (Nitsche and Paulus, 2000). Anodal stimulation depolarizes the neurons, increasing the probability of action potentials occurring, whereas cathodal stimulation hyperpolarizes neurons, thus decreasing the likelihood of action potentials occurring (Nitsche et al., 2008). These polarity-specific effects have been demonstrated in multiple paradigms (Antal et al., 2003; Priori, 2003) both during (online) and post-stimulation (offline) (see section What Are the Differences between Online and Offline Designs?).

Although tDCS is generally flexible in terms of protocols and electrical dosage, it is not easy to decide upon the most effective design for a given experiment. This is partly due to the current lack of comparable research available: there is great variability in protocol and set-up across published studies, and many of them are often under-powered due to small sample sizes (Berryhill et al., 2014; Li et al., 2015). For researchers who are new to tDCS, designing an experiment may therefore be a time-consuming process that involves sorting through many publications that lack consensus. Furthermore, only experiments yielding significant results tend to be published (Møller and Jennions, 2001) resulting in an unbalanced account of successful and unsuccessful tDCS methodologies.

This article provides a step-by-step guide on how to conduct a tDCS experiment, designed to aid researchers who are new to the technique. We highlight some basic principles that should be considered when designing an experiment and, in that process, allude to the methodological variability that may be hindering the creation of testable and evidence-based predictions. Whilst some of the guidelines we cover may be similar to those provided by the manufacturers of tDCS devices, we will also explore some equivocal issues in the literature that are not always accounted for by the “official” documentation. Furthermore, manufacturers do not always provide the most appropriate components with their devices, and we therefore hope that the advice provided here will allow new users to make more informed decisions about their paradigm.

## Why use tDCS?

tDCS is a non-invasive method, allowing for the reversible modulation of activity in particular brain regions. This has provided a valuable tool for establishing brain-behavior relationships across a variety of cognitive, motor, social, and affective domains (for a review see Filmer et al., 2014) and, in healthy populations, it has been shown to temporarily modify behavior, accelerate learning, and boost task performance (Coffman et al., 2014; Parasuraman and McKinley, 2014). For example, anodal stimulation has been shown to enhance facial expression recognition (Willis et al., 2015) or inhibit aggressive responses (Dambacher et al., 2015; Riva et al., 2015), whereas cathodal stimulation has been shown to foster implicit motor learning when stimulating the dorsolateral prefrontal cortex by suppressing working memory activity (Zhu et al., 2015). In practical terms, the equipment is reusable, relatively inexpensive, and easily replaced if worn or damaged. This contributes to its therapeutic potential in the clinical sciences—it is easy for researchers or patients to administer tDCS at home, and it may soon be used alongside (or in replacement of) drug treatments to speed recovery and improve motor and cognitive performance (Brunoni et al., 2012). Indeed, tDCS has even been successfully applied to reduce symptoms of depression (Fregni et al., 2006; Nitsche et al., 2009), although the field needs to expand further to support its use for this purpose. In small-scale studies it has been shown to reduce hallucinations in people with schizophrenia (Agarwal et al., 2013) and to improve delays of syntax acquisition in autism spectrum disorder (Schneider and Hopp, 2011).

## How do i use it?

### Performing a stimulation session

**Figure 1:**
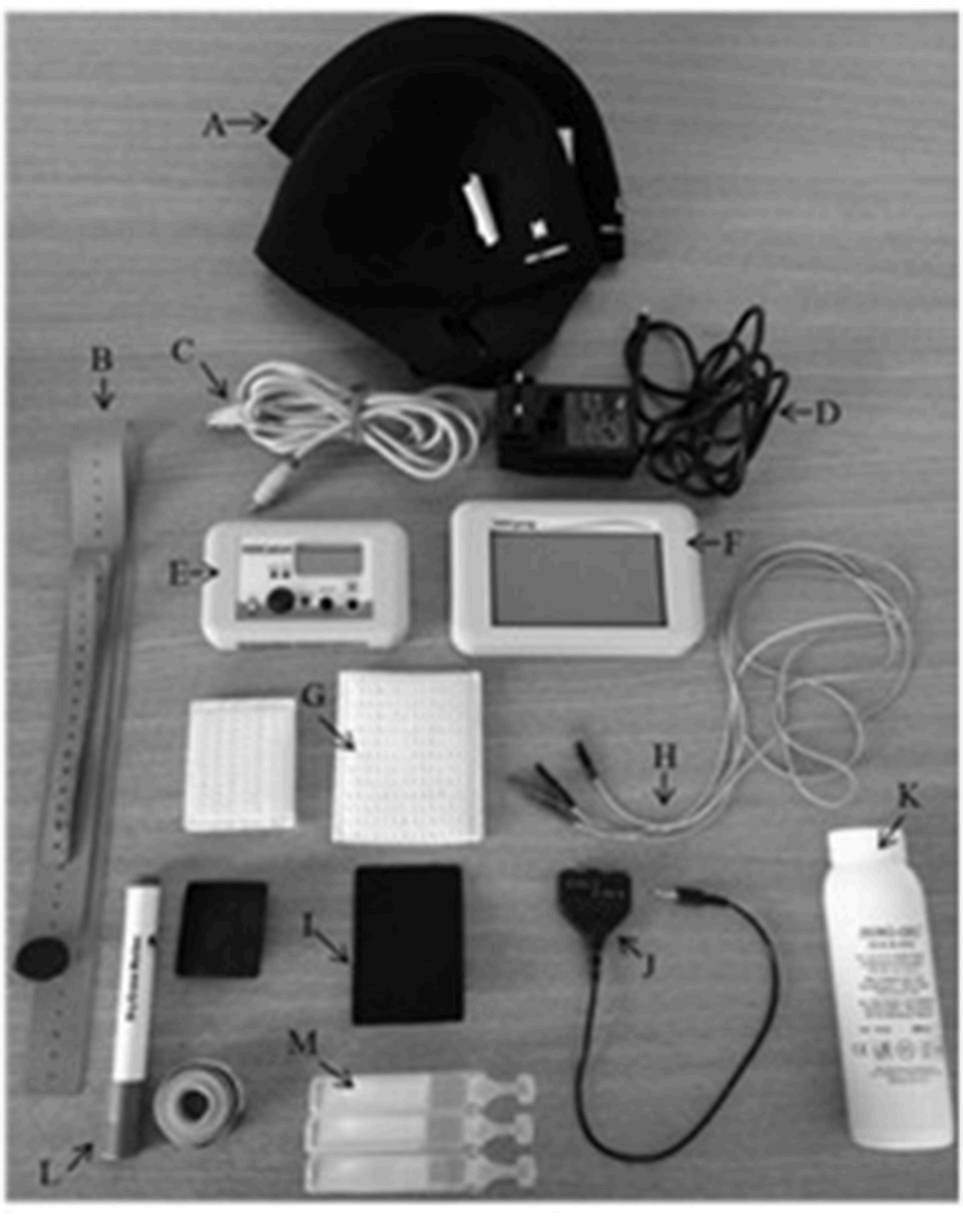
tDCS equipment for the HDC Kit. **(A)** Neoprene swimming caps for securing electrodes, **(B)** straps for securing electrodes, **(C)** programmer/stimulator connector cable, **(D)** power supply, **(E)** tDCS stimulator (batteries inside), **(F)** tDCS stimulator parameter programmer, **(G)** sponge holding bags, **(H)** electrode cables (red—anodal; black—cathodal), **(I)** rubber electrodes, **(J)** cable connector, **(K)** conductive EEG gel, **(L)** measuring equipment (washable pen and measuring tape), **(M)** saline (20 ml pouches for easy application). Not all tDCS kits come with a separate stimulator and parameter programmer.

Here we describe a standard tDCS set-up, using a target and a reference electrode. First, the desired locations of where the electrodes will be positioned need to be ascertained (further details of localization techniques are in section Localizing Electrode Placement). Prior to attaching the electrodes to the scalp, the Experimenter should ensure that there is no damaged or broken skin. If saline is being used as a conductive substance, the electrodes may be placed in sponge holding bags, saturated so that they are sufficiently damp but not dripping. However, it is becoming increasingly common to use conductive paste or EEG gel to affix the electrodes to the scalp, which may control the distribution of the current more effectively than saline. The participant's hair should be parted to ensure good contact between scalp and electrode. Saline should not run down the scalp or spread over the hair. Electrodes are then attached to the stimulator using wires connected to corresponding anodal/cathodal ports. Once the electrode is placed over the target region it should be secured using a cap, rubber bands or elastic tubular netting. The reference electrode should then be secured in the same manner. Standard apparatus are illustrated in Figure 1.

Once the electrodes have been attached, stimulation duration, current intensity, and ramp up/ramp down times need to be programmed (see section What Parameters Should I Use?). Some stimulators allow the Experimenter to pre-program the stimulation parameters while others require manual input before each session. It is important to monitor the participant during stimulation, including sham conditions, to ensure there is no discomfort experienced. It is also important to check the impedance levels displayed on the stimulator to ensure that stimulation has not failed. Reliable and consistent application of tDCS requires good contact with the scalp in order to maintain conductivity through the circuit. High impedance levels are an indicator of poor conductivity and may be the result of poor electrode set-up. Because impedance levels highlight whether the current can remain constant it is important to monitor these levels displayed on the stimulator throughout the experiment. High impedance levels can be the result of inadequate parting of the hair to allow good contact with the scalp, or a lack of conductive substance between the scalp and the electrode. DaSilva et al. (2011) recommend keeping impedance levels below 5 k ohms. A stimulation failure may therefore be resolved by reapplying saline to the holding bags, or by parting the hair beneath electrodes more sufficiently.

### Localizing electrode placement

Several methods can be used to localize the electrode placement. The most common method is the 10:20 EEG system (Klem et al., 1999). If this is used, the participant's head is firstly measured in order to accurately locate the regions of interest. This is usually done by measuring from the inion to the naison, and from the left pre-auricular to the right pre-auricular (Klem et al., 1999). Measurements can then be used in conjunction with the 10:20 EEG system to locate regions of interest. Target regions may then be signposted with a washable marker. Alternatively, neuro-navigation software can be used, which may be more accurate than the 10:20 EEG system. However, this method does depend on the participant undergoing an MRI scan. Access to past MRI scans may be achievable, but if not, it could be costly to scan each participant before undergoing tDCS. Physiology-based placement may also be used; for example, if the motor cortex is the region of interest, TMS may firstly be used to induce motor evoked potentials (MEPs) to identify this region (e.g., Nitsche and Paulus, 2000). However, physiology-based placement is currently limited to few primary cortices, meaning not all electrode localization can be dependent upon this measure (Woods et al., 2016).

Aside from the intended behavioral or physiological assay (see section What Parameters Should I Use?), it is also important to consider how the placement of electrodes will affect current flow. Modeling studies may help decide upon this, since they provide computational representations, based on realistic head models, to determine how the current may flow during tDCS (Bikson et al., 2012). Modeling studies have highlighted the importance of an individual's anatomy in current injection and flow (Miranda et al., 2006, 2009) as discussed in section What Parameters Should I Use? For example, COMETS is a recently developed MatLab Toolbox (Jung et al., 2013), that aims to assist with electrode placement, by simulating current flow amongst various electrode placements. This may be useful for new researchers to explore, but it is important to note, with any modeling study, that they are purely computational representations and that head size, shape and anatomy still varies greatly across individuals.

### Electrode contact

Saline is the most common method of ensuring conductivity with the scalp. When rating perception of comfort for different concentrations of saline, 15 to 140 mM were found to be most comfortable in comparison to 220 mM and to deionized water (Dundas et al., 2007). If impedance levels are too high, more saline can be added to the sponges to compensate, and it can be easily reapplied during stimulation whenever needed (Loo et al., 2011). However, it is important to not over-soak the sponge pouches as this can saturate hair, affecting the spread and direction of the current flow (for further discussion see: Horvath et al., 2014). Participants who have dense hair are most likely to receive over-saturated sponge pads, as the electrode-scalp contact is especially difficult to achieve (Horvath et al., 2014; Fertonani et al., 2015). We recommend using small containers of saline (such as 20 ml bottles) which allow slight control over the amount of liquid placed onto the sponges. Alternatively, electro-conductive gel (such as EEG paste) may also be used, especially for participants with thick hair. However, the use of gel will likely require participants to wash their hair after, whilst saline dries out more easily. Choosing one over the other may depend on the facilities available in one's lab, but while saline may be more common and easier for participants, it is not necessarily the best option for conductivity and secure placement with the scalp (DaSilva et al., 2011). Gels are applied to the base of the rubber electrode, so there is no need for sponge pouches. However, gel may also dry out quickly due to the temperature that the electrode emits, increasing risk of burns to the scalp (Lagopoulos and Degabriele, 2008). Note that tDCS should never be painful, although cutaneous sensations have been reported (see section Adverse Effects). One research laboratory has reported that different types of gels influenced cutaneous sensations in participants, especially viscous gels, that were also difficult to apply to the base of the rubber electrode (Fertonani et al., 2015). The use of anaesthetics applied to stimulation sites has been shown to reduce uncomfortable sensations, compared to a placebo (McFadden et al., 2011). However, their use is not advisable as they may mask the sensation of any damage being caused (DaSilva et al., 2011).

Electrodes can be secured to the scalp using rubber bands, elastic tubular netting or neoprene caps. It is highly important to ensure that the electrodes stay securely fixed in place during a stimulation—one study has suggested that as little as 5% movement can alter the accuracy and intensity of the current to the desired cortical areas (Woods et al., 2015). Most manufacturers provide rubber bands, and their advantage is that electrode placement is visible to the researcher. However, bands are usually narrower than the electrode and therefore may not ensure full contact with the scalp. Elastic tubular netting can also be used for securing electrodes, however, it is important to ensure that this material (such as cotton) does not absorb saline, as this could cause impedance errors and unwanted dispersal of the current flow across the scalp. Netting is however, easy to use and maintains uniform electrode-skin contact, by allowing the electrodes to adhere to the shape of the head (Fertonani et al., 2015). Neoprene caps are also more secure, and allow better contact with the region, although placing the electrode accurately may be slightly harder. From our own experience, neoprene caps with a chin strap are recommended.

## What parameters should i use?

### Where should i target stimulation?

The region of interest is stimulated using the target electrode, the location of which depends on the hypothesis and task. For example, if the hypothesis concerns aggression, one might focus stimulation on the prefrontal cortex (Hortensius et al., 2012). Tasks should be expected to recruit neurons in the target region, in order to observe stimulation-related changes in behavior. Bihemispheric montages (also known as “dual” stimulation) may instead be used whereby the positioning of both target electrodes is important for down-regulating one area (cathodal current) and up-regulating (anodal current) the parallel area in the opposite hemisphere. For example, if the hypothesis concerns motor outputs, one might focus dual stimulation to both motor cortices (Lindenberg et al., 2010). It is just as important in these set-ups that the target regions are recruited for the task at hand.

The target region should be on the cortical surface, as scalp electrodes do not penetrate deep brain regions. Modeling studies have demonstrated that the distribution of the current can vary across subjects, even when the electrode montage is kept consistent, due to anatomical features such as skull thickness and composition (Opitz et al., 2015). Current direction may also be influenced by lesions that may be common in clinical samples (Datta et al., 2011). Use of neuro-navigational software allows the experimenter to more accurately place electrodes above a defined cortical location, whilst taking anatomical differences across participants into account. However, researchers should be aware that no matter what method of cortical localization (see section Localizing Electrode Placement) is used, surrounding regions may receive stimulation, potentially causing unspecified changes to task performance.

### Where should the reference electrode be placed?

Placement of the reference electrode should primarily consider factors influencing the impact of its location on the task, the direction of current flow, participant comfort, and safety. Although used infrequently, some researchers have deployed montages in which two reference electrodes are positioned on the scalp (providing the same polarity), and one reference electrode is used (providing a different polarity), totaling to three, rather than two electrodes (see Nasseri et al., 2015, for further details on the classification of electrode montages). To ensure adequate stimulation in which most of the current reaches the target region, rather than being shunted across the scalp, the reference electrode is commonly placed opposite the target electrode. Some montages involve the electrodes being placed much closer together, however this should be avoided, as the current may travel through the cerebrospinal fluid (CSF) from one electrode to the other, without stimulating the cortex. This is due to the CSF being more conductive than brain tissue (Moliadze et al., 2010). Modeling research has shown that a higher percentage of current penetrates the brain if the electrodes are placed further apart (Miranda et al., 2006). A distance of at least 8 cm when using 35 cm^2^ electrodes has been recommended by a modeling study (Wagner et al., 2007). However, large distances also come at a cost, as higher stimulation intensities may be necessary (Moliadze et al., 2010). On the other hand, the current may dissipate across the scalp, meaning a decreased concentration reaches the brain region; this is known as a shunting effect. It has been suggested that if the distance between electrodes is 5 cm or less, the current would be highly susceptible to a shunting effect (Rush and Driscoll, 1968). Generally, large distances between the scalp electrodes, are expected to increase cortical modulation, allowing the current to be drawn through the cortex, rather than shunted across the scalp (Bikson et al., 2010). Additionally, smaller electrode sizes have been correlated with larger shunting effects (Wagner et al., 2007).

Electrode distance may be at its greatest if the reference electrode is placed extracephalically (not on the head), such as on the contralateral upper arm. At this location, it may be secured with hypoallergenic tape or rubber bands. One important advantage of an extracephalic electrode set-up is that it helps to exclude the effect of the reference electrode on cortical modulation, focalizing the current in the active electrode greatly (Nitsche and Paulus, 2011). However, differences in extracephalic electrode placement could cause the current direction to change; for example, switching between placement on the contralateral upper arm instead of the forearm could shift the current flow to travel across parietal regions rather than frontal (Bikson et al., 2010). Nevertheless, this concern is not necessarily unique to extracephalic placement, as differing locations of cephalic electrodes and the influence of anatomical factors can also change the current direction (Bikson et al., 2010; Datta et al., 2011).

A particularly important issue that is not always highlighted is the potential for the current to be directed toward vital areas including the heart, respiratory system and the brainstem autonomic regions (Vandermeeren et al., 2010). Initial tDCS experiments suggested that one participant experienced a short episode of respiratory depression during stimulation when the electrode was positioned extracephically on the leg (Lippold and Redfearn, 1964; Redfearn et al., 1964). However, this was using a current strength of 3 mA, which is above the present safety threshold of 2 mA (Iyer et al., 2005). More recently, a safety investigation into the effect the current has on the brainstem autonomic centers and the cardio-respiratory system, showed no significant differences in activity, during or after stimulation (Vandermeeren et al., 2010). However, only a small sample of healthy people were tested in this study, and these differences may vary in other populations, particularly those who have cardiovascular issues. Additionally, varying stimulation intensities up to the 2 mA safety threshold (Iyer et al., 2005) were not investigated, nor were a variety of electrode montages, and therefore caution is advised when considering extracephalic placement. Nevertheless, this study, and others that have investigated tDCS effects on heart rate, blood pressure, body temperature, ventilation rate and respiratory frequency (e.g., Accornero et al., 2007; Raimundo et al., 2012), provide a good indication of limited cardiac interference when using tDCS. Modeling studies have provided further insight and have shown that an extracephalic set-up does not induce brain stem interference at 1 mA (Im et al., 2012; Parazzini et al., 2014) or the heart at 2 mA (Parazzini et al., 2013). Extracephalic electrode set-ups are increasingly popular, and studies have shown significant tDCS effects using this set-up including cognitive behaviors (e.g., Axelrod et al., 2015) and psychiatric conditions (e.g., improvements in depression—Martin et al., 2011), without harm or discomfort to participants.

### What size should the electrodes be?

It is becoming common practice to have a smaller, more focal target electrode and a larger reference electrode to avoid meaningful stimulation of the reference site (see section Current Density). The most commonly used electrodes are sized between 25 and 35 cm2 (5 × 5 cm and 5 × 7 cm) (Utz et al., 2010) and the suitability of dimensions can depend on the stimulation site. More recently HD-tDCS or “ring electrodes” have been introduced (see Villamar et al., 2013, for a guide). These comprise of five small electrodes, such as a single anode surrounded by four cathodes, or vice versa (DaSilva et al., 2015). This 4 × 1 ring montage has been shown to enhance spatial focality and also overcomes problems observed when using square sponges, in which the highest concentration of current density is observed along the straight edges (Miranda et al., 2006). Furthermore, MxN stimulator systems offer the most advanced form of HD-tDCS, in that they allow the researcher or clinician to configure montages from an array of possible electrodes, allowing each to stimulate as cathodal or anodal (Rostami et al., 2013). The enhanced focality of ring electrodes is due to the suppression of surrounding regions by the other electrodes, constraining any modulation (Datta et al., 2009). Conversely, skin irritation may be increased when using ring electrodes, although this can be resolved by increasing the distance between the positive and negative electrodes, at the cost of focality (Datta et al., 2009). Thus, before deciding upon the use of HD-tDCS or traditional montages, the trade-off between focality and participant comfort should be considered.

### What stimulation intensity should be used, and for how long?

To decide which stimulation duration and intensity to use, it may be useful to replicate similar protocols that have stimulated the same target region as the proposed experiment. Over time, advocating this may naturally lead to the formation of relatively universal experimental parameters for certain behaviors, and allow much more consistent and controlled comparisons of results. Generally, most stimulation durations range between 5 and 30 min, with a current intensity between 1 and 2 mA (Bikson et al., 2009). Current strengths of up to 4 mA have been tested (e.g., in stroke patients—Chhatbar et al., 2017), although the advisable safety threshold for human studies is 2 mA (Iyer et al., 2005). Stimulation duration has been shown to modulate the length of time before cortical excitability returns to baseline levels post-stimulation (Nitsche and Paulus, 2001). For example, receiving 9 min of tDCS created after-effects of up to 30 min, whereas stimulating for 13 min increased this time to 90 min. This is important to note for both safety protocols and task timings. Furthermore, stimulation duration has also been shown to alter the effect of polarity. One study showed that after approximately 26 min of anodal stimulation, an inhibitory effect resulted rather than excitation (Monte-Silva et al., 2013). Similarly, 2 mA cathodal stimulation for 20 min has been shown to result in excitatory changes (Batsikadze et al., 2013). These studies are important as they illustrate that the effects of stimulation duration and intensity are not necessarily linear and that the relationship between these two variables requires further investigation.

### What is a Sham condition?

**Figure 2:**
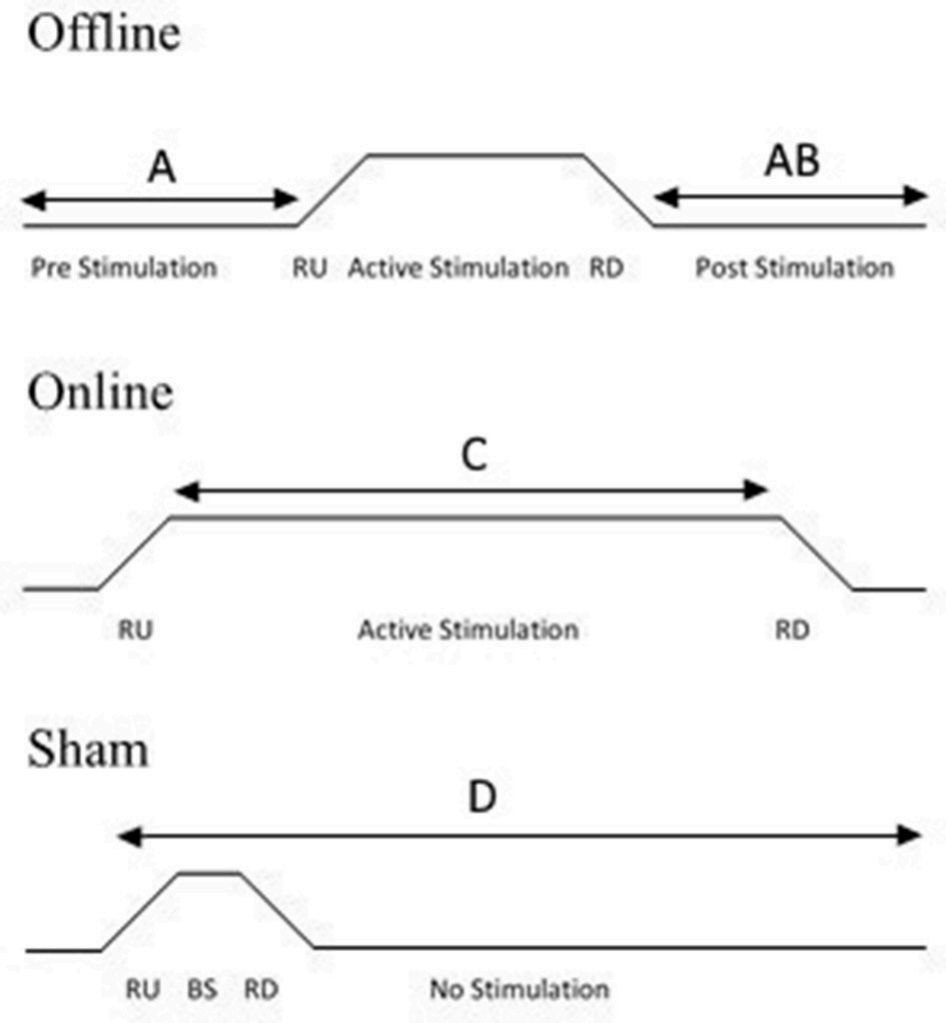
Diagram illustrating experimental protocols. Offline stimulation involves a period of pre-stimulation in which a task may be completed, followed by a period of stimulation and then a post-stimulation task **(A)** or a post-stimulation task only **(B)**. Online stimulation involves participants receiving stimulation during the task **(C)**. For sham stimulation, the task can be undertaken according to either online or offline protocols. Sham stimulation involves the current ramping up (RU), followed by a brief stimulatory (BS) period which is usually 3–5% of the active session duration, followed by a ramping down of the current. The current then remains off for the rest of the session. The task can be applied at any point during the session **(D)**, depending on whether an online or offline protocol is undertaken.

Sham tDCS acts as a control condition, in which a few seconds of stimulation at the start and the end of the programed time period is administered to a participant in order to mimic cutaneous perceptions (e.g., itching, tingling) that tend to be reported within the first few moments of the stimulator being switched on (Gandiga et al., 2006). This brief stimulation period does not change cortical excitability (Nitsche et al., 2008). Sham tDCS is easy to administer and involves three steps (see Figure 2). First, a period of “ramping up” is administered, in which the stimulator reaches the maximum programmed current (e.g., 30 s to reach 1 mA). Ramping up is then followed by a short stimulatory period, in which the participant receives stimulation for a few seconds. Finally, “ramping down” involves the current gradually being switched off. This replicates the same cutaneous sensations that are associated with changing current. There are other sham techniques, including using an alternative electrode montage that do not stimulate the region of interest (e.g., Boggio et al., 2008), or stimulating at an extremely low current (e.g., 0.1 mA with 11 cm^2^ electrode sizes) for the same amount of time as verum (“real”) stimulation (e.g., Miranda et al., 2009). However, the traditional method of ramping up/down is by far the most popular method of sham control (Ambrus et al., 2012).

Sham tDCS is generally regarded as an effective blinding technique, especially for those who have never experienced tDCS before (Gandiga et al., 2006; Ambrus et al., 2010, 2012), even at high current strengths (Russo et al., 2013). For people familiar with tDCS, blinding is more difficult to achieve and may not be overcome (Ambrus et al., 2012). Double-blind experiments are usually ideal for experimental control, however no behavioral differences have been observed between single-blind and double-blind tDCS experiments (Coffman et al., 2012) and so experimenter influences may not be as significant as expected.

### What are the differences between online and offline designs?

An online design refers to the procedure in which the participant completes the behavioral task whilst receiving stimulation. Behavioral effects can be examined during the stimulation. Alternatively, it is possible to compare the first and last “blocks” of the behavioral task in order to examine the effects of tDCS in a similar way to a pre- and post-stimulation comparison used in an offline protocol. Conversely, an offline design refers to the task and tDCS not being undertaken concurrently. An offline method can be undertaken in two ways; either the participant completes a task before and after receiving stimulation to enable a pre- and post-stimulation comparison, or the participant may only complete the task once, post-stimulation (see Figure 2 for overview of protocols). For an offline design, participants should remain inactive or complete the same control task during the stimulation period.

Although the majority of researchers broadly justify choices for certain experimental parameters (e.g., cortical location), the rationale regarding the use of online or offline methodologies is rarely presented. This decision may be based on previous studies, or may be influenced by procedural factors. For example, if the duration of the experiment must be kept to a minimum, experimenters will likely employ an online design as an offline protocol (with pre- and post- stimulation sessions) will prolong the length of the session. It may also be due to the assumption that both protocols produce the same polarity-specific outcomes (Brunoni and Vanderhasselt, 2014). However, recently the consideration that stimulation effects may be interfered with if an irrelevant activity is undertaken during, or directly after, stimulation, has been highlighted (Horvath et al., 2014) and suggests that the use of an online or an offline protocol could influence polarity specific results if an irrelevant task is completed whilst stimulation is being administered. For example, Nozari et al. (2014) found a facilitatory effect of cathodal stimulation on the Flanker task (post-stimulation) when an unrelated task was performed during stimulation. However, when participants completed a task posing the same cognitive demands as the Flanker task during stimulation, an inhibitory effect of cathodal stimulation resulted. Another example is presented by Quartarone et al. (2004), who demonstrated that motor imagery undertaken pre- and post-stimulation has different effects on the polarity of stimulation. MEP's were recorded both at rest and during motor imagery. It was found that cathodal stimulation resulted in a larger decrease in amplitude in the imagery condition than at rest. Whilst anodal stimulation was unaffected by imagery. There are many other examples of similar interference effect (e.g., Antal et al., 2007; Gladwin et al., 2012). Findings such as these should not be ignored, and show that every aspect of the procedure, including any breaks between tasks, should be planned and recorded.

Although these examples highlight the importance of control in the procedure, there may be some individual participant behaviors that are beyond the control of the experimenter such as finger tapping or other minor motor actions (Horvath et al., 2014). It is also worth considering the resting state of the target neurons prior to stimulation as this can alter the outcome (Filmer et al., 2014). Having a greater understanding of the effects of the initial brain state could help improve protocols in future perhaps through deliberate priming of the cortex (for further details see section Anodal and Cathodal Stimulation). For example, it has been demonstrated that neuronal populations that are less active respond more strongly to TMS (Silvanto et al., 2008). These findings may therefore have implications for other forms of brain stimulation, as well as tDCS, and should not be overlooked.

### What factors influence the selection of within- or between- subjects' designs?

In tDCS studies, a within-subjects design involves each participant completing all the polarity conditions, whereas a between-subjects design exposes separate groups to a single stimulation condition. A within-subjects design overcomes some of the problems of individual differences in current responsiveness (Li et al., 2015). However, in terms of the practicality of design, there are issues that must be accounted for, such as the possibility of data being confounded by learning, practice or order effects due to the repeated sessions (Berryhill et al., 2014). This can be overcome by counterbalancing the stimulation order across participants or considering practice as a factor in further analyses (Li et al., 2015). Other issues include subject attrition due to multiple testing sessions, or the potential for unspecified behavioral effects of repeated stimulation. There are currently no standardized guidelines on the amount of time that should be left between tDCS sessions to ensure that any stimulatory effects have “washed out” (Monte-Silva et al., 2010). Stimulation over consecutive days can cause cumulative and larger excitability effects (Cohen Kadosh et al., 2010; Alonzo et al., 2012; Galvez et al., 2013; Ho et al., 2015), and it is therefore advisable to leave at least a week between testing sessions (Boggio et al., 2007). It is also advisable to ensure that participants come back at the same time on all testing days to reduce the risk of circadian influences (Krause and Cohen Kadosh, 2014; Li et al., 2015).

Between-subject designs have their own pitfalls, such as masking individual differences in performance and susceptibility to tDCS (Li et al., 2015), and increasing the risk of inter-individual variability as an extraneous factor, as detailed in Table 1. Recent research has demonstrated that anodal and cathodal stimulation do not create reliable changes in cortical excitability across repeated testing sessions within the same individual (a potential pitfall of within-subject designs), however an overall increase in excitability was demonstrated at a group level for anodal stimulation. Sham stimulation was shown to have a stable effect across participants (Dyke et al., 2017). Additionally a larger sample size is required for between-subjects sub-group analysis. It may therefore be useful to report individual data to further evaluate participant variability within each polarity (e.g., Nitsche and Paulus, 2001; Horvath et al., 2016).

**Table 1:** Details of variables which can alter current flow and uptake.

**Source of variability**	**Variable**	**Citations**
Biological	Hair thickness Amount of sweat produced on the skin surface below the electrode pad	Horvath et al., 2014
	Head size Tissue thickness	Bikson et al., 2012
	Skull thickness Subcutaneous fat levels CSF density Cortical fluid density Cortical surface topography Individual morphologies of cortical gyri and sulci	Datta et al., 2012; Opitz et al., 2015
	Initial state of the cortex before stimulation	Filmer et al., 2014; Krause and Cohen Kadosh, 2014
	Neurotransmitter levels (especially GABA)	Krause and Cohen Kadosh, 2014
	Stages of the menstrual cycle	Inghilleri et al., 2004^*^
	Age	Fujiyama et al., 2014; Li et al., 2015
	Genetics (e.g., relatives of those with schizophrenia).	Hasan et al., 2013
Lifestyle	Intake of neuroaffective substances (e.g., nicotine)	Grundey et al., 2012
	Educational level	Berryhill and Jones, 2012
	Personality	Peña-Gómez et al., 2011

*This is not an exhaustive list*.

* *Inghilleri et al. (2004) found that different stages of the menstrual cycle effected cortical excitability when using repetitive TMS. These findings may be applicable to tDCS*.

### Probing tDCS effects

tDCS effects can be quantified in several ways. The most common method is indirect, via behavioral measures—i.e., researchers aim to measure whether a certain tDCS polarity modulates a given behaviors in a manner that is not usually observed under sham conditions. The positioning of active electrodes, and the choice of the task (including its associated metric), are therefore critical for the observation of tDCS effects. They may, however, be particularly subtle (Fregni et al., 2004), and so it is especially important that the task and metrics probe the specific behavior in question. The task should involve a suitable level of difficulty in order to avoid ceiling or floor effects (Fregni et al., 2004; Berryhill et al., 2014) that could be misinterpreted as tDCS effects, rather than task training effects (Woods et al., 2016). Again, this emphasizes the necessary requirement of a sham condition or baseline measure in tDCS experiments.

On the other hand, additional methodologies can be combined with tDCS to provide a more direct means to quantify cerebral changes. tDCS can be used in conjunction with techniques such as TMS, fMRI and EEG in order to examine how stimulation modulates cortical excitability. Although the focus and scope of this article is not to detail how tDCS is used with these techniques, it is still important to briefly highlight that combining neuroscience techniques may provide a superior picture of brain-behavior relationships.

The seminal effects of tDCS were measured through the application of TMS to the motor cortex and recording MEP sizes after different intensities of anodal and cathodal stimulation. It was shown that cathodal stimulation decreased MEP size from baseline, whilst anodal stimulation had the inverse effect. Change over time was also measured, showing a gradual return to baseline at approximately the same rate for both polarities highlighting continuing cerebral changes post stimulation (Nitsche and Paulus, 2000). Since then, TMS-tDCS has been used to examine causal interactions between the motor cortex and actions (Filmer et al., 2014), as well as the exploration of visual cortex excitability, by altering phosphene threshold (e.g., Antal et al., 2003).

Combining tDCS and MRI is also a fruitful avenue of research. MR Spectroscopy has been used to probe the synaptic plasticity effects of tDCS (e.g., GABA and glutamate systems) (Stagg and Nitsche, 2011). Further, fMRI can be used to examine how tDCS influences brain networks with high spatial resolution. Recent tDCS-fMRI studies have suggested that stimulation to cortical surface areas may further change the state of networked regions. For example, Hampstead et al. (2014) found that parietal-frontal tDCS altered activity in the hippocampus and caudate nucleus. This may be advantageous when considering the modulation of a network that involves deeper brain regions, however it is important to consider that without the use of fMRI to monitor these effects, the current may flow to areas that are not necessarily predicted by the researcher.

fMRI may firstly be used to identify brain regions involved in a task (baseline task). tDCS can then modulate these regions, and effects may be indirectly observed via the same behavioral task. Alternatively, fMRI may be used to explore the direct modulatory network changes after or during tDCS. Some tDCS machines are fMRI compatible, meaning online tDCS protocols can be carried out during scanning, and without the need for participants to be removed from the scanning room. Participants can therefore stay in the same position, which is advantageous when voxel placement reproducibility is necessary, or during high-resolution fMRI (Woods et al., 2016). However, integrating tDCS and fMRI may have a large financial cost, and does have many practical and safety complications. Meinzer et al. (2014) provide an extensive overview on how to conduct an fMRI-tDCS experiment, and the precautions that should be undertaken when doing so, including guidance on specialized tDCS equipment and participant considerations.

Finally, tDCS can be combined with EEG allowing for greater temporal resolution than fMRI and may further uncover a greater understanding of cortical excitability before and after tDCS as compared to TMS due to its greater spatial resolution (Schestatsky et al., 2013). So far, there have been limited studies combining tDCS and EEG (Meinzer et al., 2014). EEG can be used to examine pre- and post-stimulation cortical excitability effects of stimulation, allowing for surrogate markers of tDCS effects to be uncovered (Schestatsky et al., 2013). One system combines both EEG and tDCS electrodes into the same cap, and Schestatsky et al. (2013) provide a step-by-step guide on how to conduct a combined EEG-tDCS experiment, as well as pointers on analysis.

## Experimental and safety considerations

### Current density

Some tDCS studies use the same electrode size for both the target and reference electrodes. This set-up means that if anodal stimulation occurs at the target electrode, an equally strong cathodal current will stimulate the region under the reference electrode. To address this confounding factor, and to be confident that it is the target region stimulation that alters behavior, current density calculations (current strength divided by electrode size) can be performed in order to select a reference electrode size that would result in a level of stimulation that will not modulate cortical activity. However, current density at the skin and skull surface is always higher than current density within the brain (Wagner et al., 2007). Research has suggested that in order for stimulation to actively modulate cortical activity it should be above a minimum threshold of 0.017 mA/cm2 (Nitsche and Paulus, 2000). For example, Knoch et al. (2008) stimulated at 1.5 mA using a 100 cm2 reference electrode (current density: 0.015 mA/cm2) and a 35 cm^2^ target electrode (current density: 0.043 mA/cm^2^), resulting in an appropriate below threshold current density for the reference electrode, and above threshold for the active electrode.

It is also assumed that higher current densities translate into stronger effects, although this matter is debated. For example, Bastani and Jaberzadeh (2013) illustrated that excitability changes do not necessarily show a linear trend as current density increases. Specifically, 0.013 mA/cm2 current density had a stronger excitatory effect than 0.029 mA/cm2, however further higher densities did continue in a linear fashion. This is contradictory to the minimum threshold of 0.017 mA/cm^2^ described by Nitsche and Paulus (2000). These discrepancies in findings may be due to differences in stimulation duration (10 min, in comparison to 5 min) and electrode size (24 cm^2^, in comparison to 35 cm^2^) across both studies. When planning a tDCS study, it may be useful to examine papers that have explored different current densities and stimulation parameters.

### Anodal and cathodal stimulation

Despite relative consensus on the excitatory effects of anodal stimulation, a recent review has suggested that tDCS experiments that have stimulated non-motor regions have found limited inhibitory effects of cathodal stimulation (Jacobson et al., 2012). The same review also revealed that researchers had a 16% chance of finding polarity-specific effects. An alternative review paper also concluded that cathodal stimulation does not significantly alter cognitive function (Filmer et al., 2014). To add to the ambiguity, it has been proposed that a single session of tDCS (regardless of stimulation type) has no effect on performance (Horvath et al., 2015). Overall these differences could be due to the lack of standardized methodologies (Li et al., 2015) and the fact that not all studies administer both anodal and cathodal polarity conditions alongside a sham comparison. Indeed, a recent report suggested that approximately 90% of studies using tDCS to stimulate the motor cortex did not use a sham-controlled design (Horvath et al., 2014). Collectively these reviews emphasize the importance of including all three types of stimulation condition in an experimental design, in order to test for differing and unpredictable results.

The varying outcomes of stimulation polarity brings into question exactly how stimulation affects the target region (Dieckhöfer et al., 2006). Research examining the effect of duration and intensity of stimulation in greater detail has offered some answers, suggesting that the relationship between polarity and enhancement is highly task-dependent. For example, Antal et al. (2001) report a reduction in contrast sensitivity post cathodal stimulation but no change after anodal, perhaps illustrating an area that is already at its optimum level and therefore cannot be further enhanced. Polarity effects are also dependent on the state of each individual's cortical activity upon arrival for testing, which can be affected by a multitude of factors (e.g., alertness, caffeine intake). This can cause some participants to show facilitatory anodal effects, and others an inhibitory effect (Krause and Cohen Kadosh, 2014). Scheduling sessions at the same time each week can help ensure that a participant's routine does not interfere with polarity effects. These differences may be lost in data after averaging, but still highlight the uncertain nature of how tDCS affects underlying cortices.

### Participant factors

Published tDCS research is largely underpowered due to small sample sizes (for discussions see: Brunoni et al., 2011; Berryhill et al., 2014; Horvath et al., 2014; Shiozawa et al., 2014). Understandably, research on clinical populations may struggle to attain a large and homogenous sample. Small sample sizes can mean that detecting significant tDCS-induced behavioral effects against sham conditions may be difficult and too small to observe, or alternatively if they are significant, they may be spurious (Woods et al., 2016). Even so, power calculations can inform the appropriate sample size required for the research design. The homogeneity of a sample can also affect the reliability of results. For example, it has been suggested that anodal stimulation causes a stronger excitability response in women, compared to men (Chaieb et al., 2008), and also that men may perform more poorly on a cognitive task during cathodal stimulation (Lapenta et al., 2012). It would therefore be prudent to consider the relative representation of the sexes during recruitment.

Effects of tDCS also appear to differ depending on age, with online effects showing further enhancements in older samples (generally 55+ years) for active stimulation compared to sham. However, these increases are usually restorative rather than enhancing, due to age-related cognitive decline (Manenti et al., 2013; Zimerman et al., 2013; Fujiyama et al., 2014). As mentioned previously (see Table 1), many anatomical factors affect tDCS responsiveness, and these factors can change as the brain develops. Age should therefore be accounted for during analysis or matched as closely as possible between, or within, experimental groups.

### Adverse effects

There are no reported indications of any serious adverse effects with the use of 1–2 mA tDCS (Arul-Anandam et al., 2009). However, mild temporary side effects may occur, such as headache, a cutaneous sensation at the stimulation sites, moderate fatigue, redness of the skin under the electrode pad, difficulty concentrating, acute mood changes and nausea (Poreisz et al., 2007; Brunoni et al., 2011). These effects are self-reported within approximately 17% of healthy individuals (Poreisz et al., 2007). However, symptoms such as moderate fatigue may be related to participation in an experiment, rather than tDCS itself. The most commonly reported side effect is a cutaneous sensation (Poreisz et al., 2007), although this tends to subside once the current stabilizes (Nitsche et al., 2008). It can also be reduced by applying a moderate saline solution on the holding bag, using a ramp up/ramp down procedure when turning the tDCS on or off (DaSilva et al., 2011) and by using smaller electrode sizes (Turi et al., 2014). However, using a small electrode size may be costly for current density, as a lower current may have to be applied if current density becomes too high.

To monitor potential side effects, Brunoni et al. (2011) published an Adverse Effects Questionnaire, although only a few research groups have used the questionnaire since its publication (e.g., Manuel et al., 2014). We argue that it is advisable to take a measure of the severity of any symptoms, before and after experimentation, as well as including pseudo items (i.e., control questions) within the questionnaire. A self-report measure prior to tDCS allows the experimenter to apply discretion to judge whether an individual is fit to participate. For example, a high score on the “headache” item might indicate a painful state that could be exacerbated by stimulation. Rating symptoms after stimulation allows adverse effects associated with tDCS to be reported and for participants to be monitored if experiencing severe symptoms. See Supplementary Material (A) for an example questionnaire used by our research group.

### Exclusion criteria

With differences in experimental tasks and aims, exclusion criteria are bound to change. However, there are some commonalities across studies, and Screening Questionnaires (see Supplementary Material B) should always be used to assess any risk of participation for each individual recruited. General exclusion criteria are summarized in Table 2. It should be noted that these criteria are largely based on TMS protocols, and therefore may not all share equal relevance to tDCS paradigms (although caution is advised here).

**Table 2:** Common exclusion criteria for tDCS participant recruitment.

**Exclusion criteria**	**Reason for exclusion**
Chance of pregnancy.	Although one study has found there to be no harm to a fetus with repetitive tDCS (Vigod et al., 2014), this research is still in its early days. As a precaution, any female that believes she may be pregnant should not participate.
A history of migraines.	tDCS may cause headaches or increase the chance of a migraine attack (Poreisz et al., 2007).
If contact with the scalp is not possible (e.g., head scarf or dreadlocks).	At least one electrode must be in contact with the scalp for safety reasons and to ensure safe impedance levels.
Have a scalp or skin condition (e.g., psoriasis or eczema).	tDCS may aggravate the condition as skin is broken (Loo et al., 2011; Shiozawa et al., 2013).
Have any metallic implants, including intracranial electrodes, surgical clips, shrapnel or a pacemaker.	Means of safety. Metallic implants may also change current flow (Datta et al., 2010).
Have had a head injury resulting in a loss of consciousness that has required further investigation (e.g., a brain scan).	Head injuries may cause brain changes, meaning tDCS responsiveness and current flow may differ in this population (Datta et al., 2010).
Have had a seizure	Means of safety—seizures have been induced in similar stimulation techniques (e.g., TMS) so therefore it is advisable to exclude anyone who has previously had a seizure.
They are on prescriptive medication, or are self-medicating, apart from the contraceptive pill.	Different medications may alter seizure threshold (e.g., psychotropic drugs, Pisani et al., 2002) or alter cognitive performance (e.g., antihistamine drugs, Kay, 2000).
Have epilepsy or a history of epilepsy.	Means of safety—although there have been no reported seizures in humans during tDCS experiments, brain stimulation may alter seizure threshold, so participants with particularly sensitive seizure thresholds should be excluded (Nitsche et al., 2008).
Medical diagnoses of psychological or neurological disorders.	This may change based on the population tested, but it is important to note that participants who have a medical diagnosis of psychological or neurological disorders may be more susceptible to adverse effects (Poreisz et al., 2007; Nitsche et al., 2008) and any trauma to the brain may make the direction of current flow unpredictable (Brunoni et al., 2012)
Adverse effects to previous tDCS or other brain stimulation techniques (e.g., TMS).	Means of safety and ethics.

*Screening Questionnaires (see Supplementary Material A) should be used alongside the above criteria to screen for further exclusion requirements*.

## Conclusion

tDCS can be used to temporarily and reversibly modulate cognitive states and actions, and is an increasingly popular tool for investigating brain-behavior relationships. The aim of this article is to provide a guide for researchers who are new to the technique, and to highlight some important factors to consider during the design stage of an experiment. These factors range from recruitment practices and stimulation parameters through to the biology and lifestyle choices of participants. This can make tDCS results unpredictable, and it is therefore advisable to research different designs and thoroughly plan an experiment to control for as many variables as possible. Our current understanding of tDCS (and, indeed, this guide) may be limited by publication biases, such that experiments producing null results are unavailable for us to learn from. However, the increasing popularity of tDCS can only lead to a greater array of successful studies that are based on carefully-planned protocols. We hope that the points presented in this article will assist the reader in conducting their own successful tDCS research, and that this will lead to more work that can refine our understanding of the brain-behavior relationships.

## Author contributions

HT and AH jointly authored the manuscript and prepared it for submission. RN and AS commented on drafts of the manuscript and contributed additional text.

### Conflict of interest statement

The authors declare that the research was conducted in the absence of any commercial or financial relationships that could be construed as a potential conflict of interest.
