# Supplementary material for: Transcranial Direct Current Stimulation (tDCS): A Beginner's Guide for Design and Implementation
Source: Front Neurosci. 2017 Nov 22;11:641. doi: 10.3389/fnins.2017.00641 (PMC5702643; doi:10.3389/fnins.2017.00641)
Supplement: Supplementary file 1 [file DataSheet1.DOCX]

SUPPLEMENTARY MATERIAL A: tDCS experiment questionnaire

This questionnaire will be filled in before and after receiving tDCS. Please enter a value from 1-10, ranging from absent to severe, in the ‘Rating’ space below in response to the question: “Do any of these statements currently apply to you?” It is important that you answer all questions truthfully.

1 2 3 4 5 6 7 8 9 10

Absent Severe

| Do any of these statements currently apply to you? | Rating | | Notes |
| --- | --- | --- | --- |
|  | **Before** tDCS | **After** tDCS |  |
| 1. Headache |  |  |  |
| 1. Neck pain |  |  |  |
| 1. Back pain |  |  |  |
| 1. Blurred vision |  |  |  |
| 1. Scalp irritation |  |  |  |
| 1. Tingling |  |  |  |
| 1. Itching |  |  |  |
| 1. Increased heart rate |  |  |  |
| 1. Burning sensation |  |  |  |
| 1. Hot flush |  |  |  |
| 1. Dizziness |  |  |  |
| 1. Acute mood change |  |  |  |
| 1. Fatigue |  |  |  |
| 1. Anxiety |  |  |  |
| Others: |  |  |  |

**Instructions for application**

Before: If participants score ‘5’ or above for any of the statements*, they should not participate on the day. This is for their own safety and comfort as tDCS has been shown to temporarily aggravate some of these conditions.

After: If participants have scored ‘5’ or above for any of the statements*, they should stay in the laboratory until symptoms have subsided or until they and the researcher are satisfied for them to leave. If their symptoms persist for more than 24 hours then the researcher should be contacted and appropriate medical attention should be sought.

*Apart from questions 3, 8, 10 and 11 as these are pseudo items
