# Supplementary material for: Transcranial Direct Current Stimulation (tDCS): A Beginner's Guide for Design and Implementation
Source: Front Neurosci. 2017 Nov 22;11:641. doi: 10.3389/fnins.2017.00641 (PMC5702643; doi:10.3389/fnins.2017.00641)
Supplement: Supplementary file 2 [file DataSheet2.DOCX]

## SUPPLEMENTARY MATERIAL B: Example tDCS screening questionnaire (refer to Table 2 for details on exclusion criteria)

It is important that you answer all of the following questions truthfully.

If any of the questions/terms on this form are unclear, or if you are unsure how to answer them, please do not hesitate to ask the researcher of the study.

|  | **Yes** | **No** |
| --- | --- | --- |
| Have you ever had a seizure? |  |  |
| Have you ever had a head injury resulting in a loss of consciousness that has required further investigation (including neurosurgery)? |  |  |
| Do you suffer from migraines? |  |  |
| Do you currently have a medical diagnosis of a psychological or neurological condition? |  |  |
| Do you have any metal in your head (outside of the mouth) such as shrapnel or surgical clips? |  |  |
| Do you have any implanted devices (e.g. cardiac pacemaker, brain stimulator)? |  |  |
| Do you have a skin condition on your scalp? (e.g. psoriasis) |  |  |
| Do you have a head wound that has not completely healed? |  |  |
| Have you ever had an adverse reaction to tDCS, or any other brain stimulation technique (e.g. TMS, tRNS)? |  |  |
| For female participants: Is there the possibility that you might be pregnant? |  |  |
| Are you currently taking any prescribed medications or are self-medicating (including reactional drug use), other than the contraceptive pill? |  |  |

The possible hazards of tDCS have been explained to me, and I understand that I can withdraw at any point for any reason, and that I do not have to disclose the reason(s) to the researcher. By signing below I acknowledge that I understand this screening form and attest to its accuracy.

| **Participant's signature** | **Researcher's signature** | **Date** |
| --- | --- | --- |
|  |  |  |
